# Screening non-coding RNAs in transcriptomes from neglected species using PORTRAIT: case study of the pathogenic fungus *Paracoccidioides brasiliensis*

**DOI:** 10.1186/1471-2105-10-239

**Published:** 2009-08-04

**Authors:** Roberto T Arrial, Roberto C Togawa, Marcelo de M Brigido

**Affiliations:** 1 Biology Institute, Molecular Biology Laboratory, University of Brasília, Brazil; 2 Bioinformatics Laboratory, Embrapa Genetic Resources and Biotechnology, Brazil

## Abstract

**Background:**

Transcriptome sequences provide a complement to structural genomic information and provide snapshots of an organism's transcriptional profile. Such sequences also represent an alternative method for characterizing neglected species that are not expected to undergo whole-genome sequencing. One difficulty for transcriptome sequencing of these organisms is the low quality of reads and incomplete coverage of transcripts, both of which compromise further bioinformatics analyses. Another complicating factor is the lack of known protein homologs, which frustrates searches against established protein databases. This lack of homologs may be caused by divergence from well-characterized and over-represented model organisms. Another explanation is that non-coding RNAs (ncRNAs) may be caught during sequencing. NcRNAs are RNA sequences that, unlike messenger RNAs, do not code for protein products and instead perform unique functions by folding into higher order structural conformations. There is ncRNA screening software available that is specific for transcriptome sequences, but their analyses are optimized for those transcriptomes that are well represented in protein databases, and also assume that input ESTs are full-length and high quality.

**Results:**

We propose an algorithm called PORTRAIT, which is suitable for ncRNA analysis of transcriptomes from poorly characterized species. Sequences are translated by software that is resistant to sequencing errors, and the predicted putative proteins, along with their source transcripts, are evaluated for coding potential by a support vector machine (SVM). Either of two SVM models may be employed: if a putative protein is found, a protein-dependent SVM model is used; if it is not found, a protein-independent SVM model is used instead. Only *ab initio *features are extracted, so that no homology information is needed. We illustrate the use of PORTRAIT by predicting ncRNAs from the transcriptome of the pathogenic fungus *Paracoccidoides brasiliensis *and five other related fungi.

**Conclusion:**

PORTRAIT can be integrated into pipelines, and provides a low computational cost solution for ncRNA detection in transcriptome sequencing projects.

## Background

Proteins are recognized as the most important players in cell homeostasis. Due to their importance and relatively straightforward characterization, it is expected that the main focus of transcriptome projects will be transcripts that code for proteins. To meet this demand, several specific computational tools have been created, both for absolute characterization and comparative analysis of these molecules. Only recently has attention begun to turn to those transcripts ignored or rejected by protein-oriented software packages: the so-called non-coding RNAs (ncRNAs). Classical, textbook examples of ncRNAs include ribosomal and transfer RNAs. More recently, other classes have been unveiled, such as microRNAs, siRNAs, piRNAs, asRNAs and the long, mRNA-like ncRNAs, widespread among all Domains, with evidence of ubiquitous tissue expression in plants and animals [1,2].

Demand is now arising for specific tools for working with these molecules. A combination of new computational tools and advances in biological knowledge allowed for development of specific software for this purpose [3]. Currently, it is not difficult to find software designed for the identification and characterization of individual ncRNA classes (as we will discuss later). However, the task is still considered complex and remains an open topic in bioinformatics.

Machine learning algorithms represent a solution for highly accurate detection and characterization of ncRNA patterns, and more improvements are expected as ncRNA biological properties are determined by biochemical and molecular experiments. Successful implementations have been reported for siRNA [4] and miRNA [5]. The mRNA-like ncRNA, on the other hand, is arguably a class which is harder to identify due to its resemblance to mRNA molecules: they may be capped, may undergo splicing, and even harbor polyadenylation and ORF signals [6]. Screening of mRNA-like ncRNA is possible on prokaryotic genomes using RNAGENiE [7]. For transcriptome contexts, there are two notable implementations: CONC [8] and CPC [9]. Both algorithms – CONC and CPC – can distinguish mRNA from ncRNA with high accuracy. CONC showed that putative proteins from ncRNA are distinguishable from those translated from mRNA, and CPC improved this idea by heavily focusing on homology information. However, their high accuracy relies on the quality of homology information (especially CPC), and both expect full-length sequences given the ORF translation schemes employed (especially CONC). These two assumptions hinder the use of these programs for analysis of transcriptomes from poorly characterized organisms because many of their sequences lack known protein homologs and are commonly built from low-quality, single-pass reads. Such drawbacks require special procedures to be employed for accurate analysis because canonical translation signals are often missing. The result is a bias toward false negatives when the input consists of low quality sequences because most transcripts code for unusual or truncated (but functional) proteins. Moreover, despite advances reported on CPC, the required computational processing power and running time remain prohibitive for labs with limited budgets.

In summary, these programs may be inappropriate for transcriptomes from neglected species. We propose new Support Vector Machine-based software to overcome these obstacles. EST sequencing errors, frameshifts and truncations are taken into account and corrected by a specially designed program, and a shunt is imposed on sequences without a predicted ORF, which are then analyzed separately. Database representation bias is eliminated by avoiding homology information and using only *ab initio *features. Also, only computationally light programs were chosen for calculation of features so as to allow pipelining from transcriptome sequencing projects with less demands on computational processing power.

## Implementation

### Putative EST translation

The ANGLE software package [10] was chosen for translation of ESTs because it focuses on sequencing errors of the input sequences and has superior performance when dealing with small sequences. ANGLE implements a hybrid method composed of a sliding window CDS classifier using a weak learner, a hidden Markov model coupled to dynamic programming for determining optimal ORF path and a frameshift detector. The dynamic programming (DP) algorithm evaluates and punctuates putative proteins translated from the six frames; among all alternatives, the putative ORF with highest DP score is taken as the protein product coded by the transcript. Transcripts are separated into two groups: those with translated proteins and those that lack any putative ORF. A user-friendly interface for ANGLE was developed in PERL and is available from the authors upon request.

### Support Vector Machines settings

Support Vector Machines (SVM) is a state-of-the-art machine learning algorithm developed from a solid statistical basis [11]. SVMs have been shown to be successful and useful in Bioinformatics [12] and several other fields [11].

We used the LIBSVM v2.84 implementation [13] with Radial Basis Function kernel, which was shown to be the best kernel to deal with this task (Liu et al, 2006), set as C-SVM and binary classification problem, with the two classes being coding (positive set) and non-coding (negative set) RNA. Optimization of parameters (C and gamma) occurred in two runs using the accompanying *grid.py *script with 20,000 randomly selected instances from the main training set. Two models were induced separately: a protein-dependent one induced with dbTR_OP as training data, and a nucleotide-only using dbTR_OA for training [see Additional file 1].

### Compared programs settings

PORTRAIT was benchmarked against two other classification programs: Naïve Bayes and CPC. Naïve Bayes (nB) is a machine learning algorithm used when a wealth of examples (or instances, or realizations) of a random variable is available, and it is desired to induce a model that is able to explain the distribution of this data. This induced model may be used to classify data yet unseen by the classifier. Although very simplistic, nB is also known to be fast and reliable, sometimes even surpassing more sophisticated machine learning algorithms [11].

Bayesian models were induced using the software package BC [14] with default parameters. Training was done with the same sets, features and normalization schemes used for SVM.

CPC [9] was installed locally and always executed with default parameters. CPC comes pre-installed with a classification model developed by its authors, which was developed using the database created by the authors of CONC [8].

### Efficiency measures

Efficiency formulas, points for plotting ROC curves and area under ROC curves were calculated both by using PERL scripts and the PERF software [15].

Cross-validation is a traditional machine learning technique for estimating classifier performance by splitting the training set into *n *equally-sized datasets, without element repositioning. Afterwards, each subset is trained once and the model is evaluated on the *n-1 *remaining subsets. This process is repeated *n *times so that each subset is used for training exactly once. We used ten-fold cross-validation, which was carried out using LIBSVM for SVM, and a custom PERL script for naïve Bayes.

### Fungal sequences

EST sequences of organisms phylogenetically related to *P. brasiliensis *(*Ajellomyces capsulatus*, *Aspergillus niger*, *Saccharomyces cerevisiae, Schizosaccharomyces pombe *and *Cryptococcus neoformans *var. *neoformans*) were downloaded from the Entrez Nucleotide Database [16] and stored as FASTA-formatted files. After filtering transcripts shorter than 80 and longer than 65,335 letters, these sequences composed the dbFG set, comprising 137,629 entries.

## Results and discussion

### Training set construction

Seeking to discriminate between ncRNA and mRNA, we used Support Vector Machines (SVM) for induction of a classification model. SVM is a supervised machine learning method, and as such, it requires previously labeled data – the training set – for model induction (see Methods for details). In this work, mRNAs (the positive set) and ncRNAs (the negative set) compose the SVM training set, called dbTR (TRaining DataBase).

#### Protein-coding transcripts

**Figure 1:**
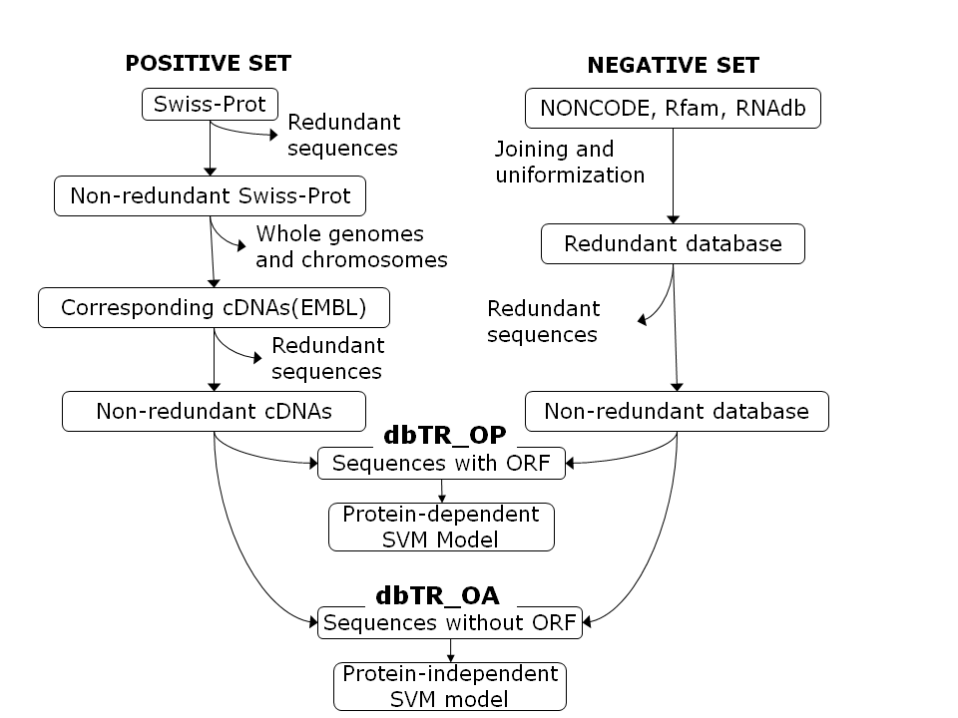
**Construction of the training database (dbTR)**. The dbTR comprises both negative and positive instances, and was subdivided as transcripts having identified ORFs (dbTR_OP) and transcripts lacking ORFs (dbTR_OA). Each of these subsets harbor their own negative and positive instances. dbTR_OP training subset was used to induce the protein-dependent SVM model, while dbTR_OA training subset generated the protein-independent SVM model.

This set was built from the SwissProt sequences file [17] downloaded in October 2006 (release 50.8), containing 234,112 protein sequences with high quality annotations. Redundancy was eliminated by using CD-HIT [18] with parameter c = 0.7. Unique accession codes were parsed and used to retrieve corresponding cross-referenced mRNA accession codes from EMBL [19], when present. Parsed IDs were screened to remove repeated entries, references to whole genomes/chromosomes or invalid entries, and subsequently used to retrieve corresponding FASTA sequences through the EBI Dbfetch service [20]. Sequences longer than 65,535 or shorter than 80 letters were excluded to avoid software incompatibility. Also, this size filter eliminates many of the so-called structural ncRNAs, like siRNAs, miRNAs, among others. Closely related nucleotide sequences were clustered using BLASTCLUST [21] with parameters S = 0.5, L = 0.5 and W = 18. Clustering at both amino acid and nucleotide levels is an attempt to eliminate over-representation of frequent homologs and protein families. Protein prediction was carried out with ANGLE software [10]. Transcripts harboring a predicted ORF and their respective protein products were assembled in the dbTR_OP training set, comprising a total of 55,372 sequences. Those transcripts lacking ORFs were assembled in the dbTR_OA set. The whole process of positive set construction is illustrated in the leftmost part of Figure 1.

#### Non-protein-coding transcripts

Files containing sequences from RNAdb [22], NONCODE [23] and Rfam [24], currently the three most comprehensive ncRNA databases, were downloaded in October 2006, comprising a total of 213,849 sequences. Nucleotide redundancy removal was done using BLASTCLUST with L = 0.5, S = 0.5 and W = 18. ORF prediction and redundancy elimination was carried out in the same way as in the positive set. The resulting 70,667 transcripts with ORFs and corresponding proteins integrated the dbTR_OP set, while remaining transcripts were merged into the dbTR_OA set. This process is shown on the rightmost part of Figure 1.

#### Feature vectors

Training and testing sets must be input as numerical values to the SVM. Properties, or features, which represent sequences as values are collectively called the feature vector. Qualitative properties are quantified, and all properties are individually normalized to real (continuous) numbers in the range (0, 1). All features were obtained using locally written Perl scripts, except for compositional entropy. Normalization procedures, the number of allocated variables and references supporting each feature's discrimination potential [25-31] are shown in Table 1 [see Additional file 2].

**Table 1:** Feature vector description. Cited references either support the coding/non-coding discrimination power of the feature or describe the corresponding program.

**Feature**	**# variables**	**Normalization method**	**Ref**.
Nucleotide composition*	84	Individual nucleotide frequency divided by total nucleotide frequency	[25]
Transcript length**	4	Binary coding: length intervals < 100, 400, 900 and > 900.	[26]
Amino acid composition^§^	20	Individual amino acid frequency divided by total amino acid frequency	[27]
ORF length^§^	4	Binary coding: length intervals < 20, 60, 100 and > 100.	[28]
Isoelectric point^§^	1	Value divided by 14	[29]
Compositional entropy^§^	1	Amount of low complexity residues divided by sequence length	[30]
Mean hidropathy^§^	1	Summed means from sliding 3nt window	[31]

*Includes nucleotide, dinucleotide and trinucleotide composition.

**Used only on protein-independent models.

§ Used only on protein-dependent models.

#### SVM optimization, training and testing

dbTR_OP and dbTR_OA were further randomly sub-divided on optimization, training and testing subsets, comprising, respectively, 20,000, 30,000 and 23,976 instances for dbTR_OP, and 10,000, 20,000 and 22,002 instances for dbTR_OA. Optimization set was used to obtain the best pair of values for two crucial SVM Radial Basis Function (RBF) Kernel parameters, the gamma and C, determined from a 10-fold cross-validation grid search. Training sets were used to induce SVM models, and test sets (from now on called dbTS_OP and dbTS_OA) were used to estimate performances of induced models.

### Efficiency measures

Estimations of model performance are evaluated by traditional methods, such as efficiency formulae, cross-validation, ROC curves and running time comparison between related programs.

#### Efficiency formulas and runtime comparisons

For estimation of classifier accuracy, we used cross-validation (CV) with dbTR_OP and dbTR_OA as training/testing sets. Figures obtained for PORTRAIT and naïve Bayes (nB) were compared to those reported in the literature for CPC.

When only the protein-dependent model is used, PORTRAIT presents a CV accuracy of 93.3%, while the CV accuracy for PORTRAIT using solely the protein-independent model is 90.9% (results not shown). Looking at the CV results from PORTRAIT running with both models, however, it had slightly worse accuracy than CPC (Table 2 – see "CV acc."). On the other hand, PORTRAIT performs comparably to CPC (see F-measure) when used for prediction of dbTS_OP and dbTS_OA (Table 2 – see measures other than "CV acc."). CPC has higher accuracy for identification of mRNAs (sensitivity), while PORTRAIT correctly identifies a greater amount of ncRNAs (higher specificity). The lightweight programs used by PORTRAIT result in a considerable speed improvement, as can be seen in Table 2. As expected, the nB classifier had worse performance but yielded a speed advantage over the SVM-based classifiers (PORTRAIT and CPC).

**Table 2:** Speed performance (in minutes), standard efficiency measures and cross validation accuracy. Indices were calculated from the mean of predictions of the classifiers regarding dbTS_OP and dbTS_OA sets.

	ACC (%)	SPC (%)	SEN (%)	F-M (%)	PPV (%)	Time (min.)*	CV acc. (%)
PORTRAIT	91.9	95.6	86.5	90.8	92.9	21.6	92.1
nB classifier	73.2	75.1	70.1	72.6	65.3	16.1	72.9
CPC	90.8	90.9	90.7	90.8	87.0	1,789.7	95.8**
Random	49.1	45.6	54.6	49.7	44.0	0.07	-

ACC = accuracy; SPC = specificity; SEN = sensitivity; F-M = F-measure; PPV = positive predictive value; CV acc. = cross-validation accuracy. Prediction threshold in all measures was 0.5 for all classifiers.

*All processes were run on a Intel^® ^XEON™ 1.80 MHz × 86 with 512 Mb RAM computer. Runtime refers to prediction of 6,022 sequences from dbPB.

**As reported by Kong et al, 2007. Training set used was not dbTS.

#### ROC curves

**Figure 2:**
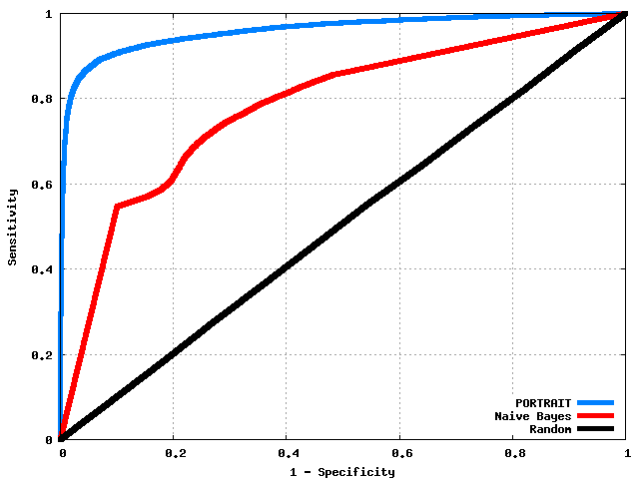
**ROC curves showing performance of classifiers on dbTS sets**. Sensitivity is plotted against (1-specificity), allowing accuracy comparisons among classifiers. A perfect classifier would yield a curve with a point at (0,1) and the final point in (1,1), that is, top-leftmost curves have better classification performance. Classification threshold was set to 0.5 for all classifiers.

ROC curves (Figure 2) estimated from prediction of dbTS_OP and dbTS_OA corroborate PORTRAIT's superior performance when compared to nB. The area under curve (AUC), an efficiency measure calculated from ROC curves ranging from 0 (minimum accuracy) to 1.0 (maximum accuracy), is 0.96 for PORTRAIT, 0.78 for naïve Bayes and 0.50 for Random classifier. CPC and CONC were excluded from ROC analysis because of output score normalization issues and the long time taken for processing dbTS sets, respectively.

#### Test sets

Induced classifiers were used to evaluate the coding potential of transcripts from three test sets. The first one is dbRD, comprising 3,000 randomly generated transcripts with lengths varying from 80 to 3,000 nt. Another set is dbPB, which harbors 6,022 assembled ESTs generated during transcriptome sequencing of the pathogenic fungus *Paracoccidioides brasiliensis *[32]. The third set is dbFG, composed of 137,629 transcript sequences from organisms phylogenetically related to *P. brasiliensis*: *Ajellomyces capsulatus *and *Aspergillus niger*, and as outgroups, *Saccharomyces cerevisiae, Schizosaccharomyces pombe *and *Cryptococcus neoformans *var. *neoformans*.

The number of transcript sequences from the test sets predicted to be ncRNA by the classifiers is shown in Table 3.

**Table 3:** Proportion of transcripts predicted as ncRNA by three classifiers.

	**dbPB**	**dbRD**	**dbFG**
PORTRAIT*	15.8%	60.5%	26.4%
Naïve Bayes*	3.2%	14.6%	12.1%
CPC**	33.1%	100%	49.8%

*A transcript is considered ncRNA if its prediction score is below 0.5.

**A transcript is considered ncRNA if it is labeled as "noncoding" by the program.

### Analysis of dbPB transcripts classified as ncRNA

**Figure 3:**
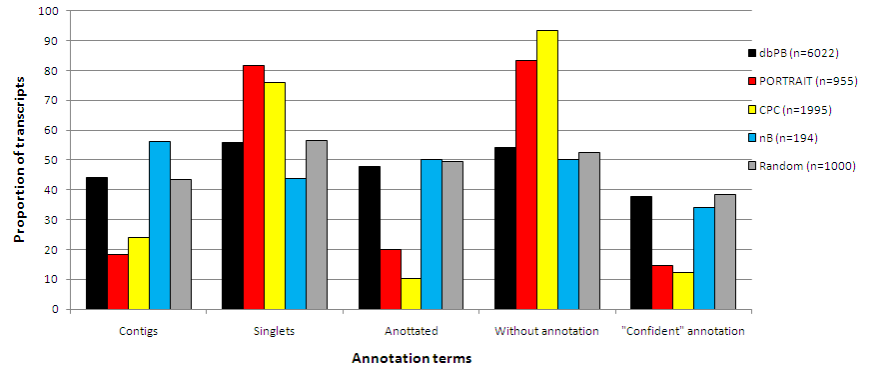
**Distribution of *P. brasiliensis *transcript sequences classified as ncRNA by several classifiers as a function of specific annotations by Felipe et al. (2005)**. Annotations of the 6,022 transcripts [32] were considered only after classifier prediction, so even transcripts previously manually annotated as proteins were evaluated for coding potential. A "Confident annotation" refers to a transcript description which lacks the words: "*putative*", "*probable*" and "*hypothetical*". The numbers of transcripts classified as ncRNA are shown in the legend (except for dbPB, which shows the total of Pb transcripts).

Next we examined among the 6,022 transcripts from the *P. brasiliensis *assembled ESTs those which were classified as non-coding by nB, PORTRAIT and CPC, all with default parameters. The dbPB set already has annotations attributed to its sequences, which were determined during a project that sequenced its transcriptome [32]. During assembly phase, transcripts assembled from more than one EST were regarded as *contigs*, but if no consensus was generated from the sequence, it was named *singleton*. We assessed whether this set of potential ncRNAs was enriched for specific annotation terms, as well as contigs or singletons. Figure 3 shows that all classifiers tend to select as ncRNA those transcripts without annotation, and that those with annotations possess terms that may question the correctness of their descriptions. These terms were: "*putative*", "*probable*" and "*hypothetical*". Moreover, the majority of these sequences are singletons.

Using the dbPB EST, PORTRAIT classified 16% as potential ncRNAs, 83% of those being unannotated sequences, thus presenting parallel evidence that those transcripts may not indeed code for proteins [see also Additional file 3]. It is important to note that this result corroborates non-coding status as an independent diagnostic only for PORTRAIT and nB, because these are the only *ab initio *classifiers. Additionally, 81% of the transcripts predicted as ncRNAs were singletons, corroborating evidence that ncRNAs are expressed at levels lower than mRNAs and thus tend to be assembled as singletons [1].

## Conclusion

In this work we report an algorithm for identifying non-coding RNAs in a transcriptome context. The distinguishing characteristic of our approach is the focus on non-model organisms: by using an ORF translation program sensitive to low-quality EST sequences, and also by choosing only *ab initio *features. Even if the input sequence has been disrupted by frameshifts or indels to an extent where ORF identification is compromised, still the query transcript may be classified as protein-coding by the protein-independent SVM model of PORTRAIT. Therefore, our predictions are not biased to classify as ncRNA transcripts that may actually code for novel proteins, rare or even absent in the databases. This may be a factor contributing to the high specificity of PORTRAIT (Table 2). Also, our training set includes several recent ncRNAs and mRNAs from all life Domains, including prokaryotic and eukaryotic sequences. These factors make our program ideal for analysis of neglected or poorly characterized species.

Differences from the *ab initio *approach also show up in the number of transcripts predicted to be non-coding in comparison to the other classifiers (Table 3). Compared to SVM, the nB algorithm is notably less complex and less robust to inconsistencies in the training set. Thus, when looking at the number of predicted ncRNAs in the dbPB and dbFG sets, one may infer that the rules derived by this algorithm for identifying ncRNAs are far too simple, leading a significant amount of ncRNAs to be misclassified as mRNA (too many false positives). On the other hand, CPC classifies all transcripts from dbRD as being non-coding. At a glance, this result seems consistent; however, some of these randomly generated sequences could be "real" mRNA transcripts encoding for novel proteins not found in the databases (false negatives). This scenario is plausible for sequences from the transcriptomes of neglected organisms, for which very little is known and where there is the potential for novelty. Taking this hypothesis into account, CPC may not be suitable for this situation because it may be biased for classifying as non-coding those transcripts lacking good hits from protein databases. PORTRAIT emerges as a compromise between nB and CPC: it predicts as ncRNA a reasonable number of the transcripts from dbPB and dbFG, and also classifies some dbRD transcripts as mRNA, despite not having come into contact with similar sequences in the training phase.

We propose PORTRAIT, a software for ncRNA screening in transcriptomes. Our method is tailored to the analysis of neglected organisms: 1) we use a 6-frame translation scheme that takes into account sequencing errors and is optimized for small or truncated sequences; 2) no homology information is used; 3) only lightweight programs are used, so the method is suitable for less powerful computers. The output of the program may also provide insights or a second opinion about the coding status of known protein-coding transcripts. Subsequent homology analyses are up to the researcher and constitute an independent, parallel experiment.

## Availability and requirements

• **Project name: **PORTRAIT

• **Project home page: **

• **Operating system(s): **LINUX

• **Programming language: **PERL

• **Other requirements: **LIBSVM 2.84, CAST 1.0, ANGLE

• **License: **GNU GPL

• **Any restrictions to use by non-academics: **PORTRAIT is free for commercial use, but third-party authors of programs used by PORTRAIT must be contacted.

## Authors' contributions

MMB designed the methods for the study and coordinated the work. RTA performed data collection and processing, algorithm implementation and interpretation of results under the supervision of MMB and RCT. RCT managed software execution, data integrity and webserver construction. RTA wrote most of the manuscript, which was critically reviewed by all authors. All authors read and approved the final manuscript.

## Supplementary Material

Additional file 1 **PORTRAIT standalone package**. For working properly, PORTRAIT still needs the following third-party softwares: LIBSVM v2.84, CAST v1.0 and ANGLE. Click here for file

Additional file 2 **SVM Feature weights**. The one hundred and fourteen variables and respective discrimination scores. Click here for file

Additional file 3 **List of transcript sequences identified as ncRNA, along with individual confidence scores**. Annotations attributed by Felipe et al. (2005) are included in sequence description. Click here for file
